# Time cannot heal all wounds: Wealth trajectories of divorcees and the married

**DOI:** 10.1111/jomf.12824

**Published:** 2022-01-29

**Authors:** Nicole Kapelle

**Affiliations:** ^1^ Department of Sociology University of Oxford Oxford UK; ^2^ Nuffield College University of Oxford Oxford UK; ^3^ Leverhulme Centre for Demographic Science (LCDS) University of Oxford Oxford UK

**Keywords:** divorce, economic well‐being, gender, inequalities, longitudinal research, remarriage

## Abstract

**Objective:**

To explore disparities in wealth trajectories between divorcees and continuously married individuals including moderation effects of remarriage and gender.

**Background:**

Amid concerns of long‐term economic consequences of divorce, research illustrated that ever‐divorced individuals hold less wealth than the married preretirement. However, it remains unclear whether this is a direct result of immediate, lasting divorce‐related wealth penalties or whether divorce also leads to long‐term wealth accumulation disparities.

**Method:**

Using personal‐level, longitudinal wealth data from the Socio‐Economic Panel Study, I applied propensity score and exact matching with random‐effects growth models to compare wealth trajectories of divorcees and the married. The matching allowed (1) married controls to be assigned a theoretical divorce date for ease of comparability to the treatment group (i.e., divorcees) and (2) the account of a wide range of baseline differences.

**Results:**

Wealth differences between ever‐divorce and continuously married individuals stem from lasting disadvantage—particularly for housing wealth—generated immediately around divorce rather than a scarring of divorcees' wealth accumulation. Remarriage but particularly gender is relevant moderators. Whereas remarriage moderates net wealth trajectories through housing wealth, gender moderates trajectories through financial wealth.

**Conclusion:**

Divorce importantly contributes to wealth stratification. Mitigation of divorce‐related wealth penalties for both men and women needs to focus on immediate, but lasting costs of divorce particularly regarding homeownership.

## INTRODUCTION

Amid historically high divorce rates across Organisation for Economic Co‐operation and Development (OECD) countries and a rising relevance of individuals' access to sufficient private wealth to secure living standards throughout the life course (Eurostat, 2018; Keister & Moller, 2000), concerns have been raised about potentially long‐term economic consequences of marital dissolution for men's and women's wealth (i.e., assets minus liabilities). Following these concerns, a small body of predominantly US‐based cross‐sectional research unequivocally found that ever‐divorced men and women held substantially lower wealth in late working age compared to continuously married men and women (e.g., Wilmoth & Koso, 2002; Zissimopoulos et al., 2015). Thus, divorce likely contributes to rising between‐household wealth inequalities.

Previous research is limited in two ways that hamper our understanding of how divorce stratifies wealth. First, previous studies commonly relied on cross‐sectional methods considering wealth disparities at a single point in time in older age. Theoretically, studies discussed older‐age wealth disparities between the married and ever‐divorced as a result of (1) divorce‐related immediate wealth penalties and (2) long‐term wealth accumulation disparities. However, an empirical exploration of these two aspects was methodologically unfeasible. Thus, I argue that a longitudinal empirical strategy is needed to understand how divorce is linked to a disruption of wealth trajectories over time. A thorough understanding of divorce‐related wealth stratification is relevant to researchers and policymakers alike to discuss how wealth disparities across marital states can be minimized and ensure economic self‐sufficiency amid growing family complexities.

Second, due to data limitations, previous studies predominantly focused on household‐level wealth. To compare between continuously married and ever‐divorced respondents, researchers divided household wealth by two for couple households (i.e., per capita wealth). Thus, studies implicitly assumed that household wealth is owned equally during the marriage and split equally in the case of divorce. However, not all resources are split equally at divorce with premarital wealth or inheritances and gifts received during the marriage regularly excluded from the division process (Kapelle & Baxter, 2021). Additionally, although long‐term married spouses share and pool a substantial proportion of their resources, wives commonly hold less personal wealth than their husbands with independent money management particularly prevalent in remarriages (Burgoyne & Morison, 1997; Kapelle & Lersch, 2020). Thus, assumptions about wealth division within (re)marriage and at divorce may have biased and limited previous research particularly with regards to potentially moderating effects of remarriage and gender.

In the present study, I used unique longitudinal, *personal*‐level wealth data collected in survey years 2002, 2007, 2012, and 2017 from the German Socio‐Economic Panel Study (SOEP v36; 1984–2019) to address the outlined shortcomings. More precisely, I answered two research questions: (a) How do wealth trajectories of divorcees differ from wealth trajectories of continuously married individuals? And (b) How do remarriage and gender moderate these trajectories? Defining wealth trajectories as unique wealth accumulation pathways that unfold over time, I utilized random‐effects growth models to empirically explore how immediate divorce penalties and potentially deteriorated wealth accumulation up until 30 years after divorce explain previously highlighted wealth differences between the continuously married and ever‐divorced. In preparation of the outcome regression for which I predicted trajectories of *untransformed* personal wealth data for divorcees and the married, I matched divorced and continuously married respondents on predivorce differences using a combination of (coarsened) exact and nearest neighbor propensity score matching. The matching provided two relevant advantages: (1) it more appropriately accounted for selection into divorce in the outcome regression. (2) The matching provided a way to systematically assign a hypothetical divorce date to continuously married sample respondents. Thus, a common time scale could be generated for ease of comparability between divorcees and the married.

## HOW DIVORCE MAY DISRUPT WEALTH ACCUMULATION TRAJECTORIES

Wealth trajectories naturally differ between individuals due to unique opportunity structures and constraints of financial decision‐making based, inter alia, on educational achievements, occupations, race, family of origin characteristics, or gender (Killewald et al., 2017). Additionally, the anticipated wealth trajectory (i.e., on average rising wealth levels throughout the working life) can be substantially disrupted and altered through certain life course events, often denoted “turning points” (Abbott, 1997). Marital dissolution may be considered such an event, or shock, that has not only the potential to immediately and drastically change the wealth levels around the event, but to also permanently alter the conditions of gaining or maintaining wealth in the future compared to continuously married individuals.

### Initial level effect: Immediate wealth level disruptions associated with divorce

Marital dissolution is likely associated with distinct, immediate changes in wealth levels due to increased financial demand and a range of wealth‐relevant burdens (Kapelle & Baxter, 2021; Zagorsky, 2005). First, direct expenses of the divorce proceedings relate to administrative divorce costs (i.e., court fees and solicitor fees) which commonly increase with the complexity of the divorce case and value in dispute. In the United States, administrative divorce costs can easily exceed the yearly household income of the former couple (Henry et al., 2011). In Germany, administrative divorce costs (i.e., court fees and solicitor fees) are legally stipulated and can start from under €1000 for childless spouses with low values in dispute. However, court fees increase with the case's complexity, and solicitors' fees are not legally capped. Thus, administrative divorce costs can be substantial. Second, legal divorce requires the division of marital assets (i.e., wealth accumulated during the marriage commonly excluding personal inheritances and gifts). Whereas some assets may be easily divided, such as savings in a bank account, other assets can be indivisible and liquidation may be necessary. This is particularly likely for the family home, which is commonly jointly owned and constitutes the major share of the marital wealth portfolio (Thomas & Mulder, 2016). As spouses often lack sufficient cash collateral to buy out the other partner or are unable to qualify for a mortgage by themselves, housing property is regularly sold when spouses divorce (Mikolai et al., 2019). Property sales incur direct costs such as notary and real estate fees, or early repayment charges for premature terminations of mortgage contracts. Within the German prudential mortgage system these costs are particularly high within international comparison. Additionally, property sales—but also sales of other assets such as shares—may be associated with indirect costs of wealth depreciation if assets need to be sold under time pressure and in a market unfavorable to the seller. Third, requirements for legal divorce often force at least one spouse to relocate before the divorce proceeding to demonstrate physical separation of spouses. As living in the formerly marital home without the partner can be costly, budget constraints often force both spouses to eventually relocate to a more affordable dwelling (Mikolai et al., 2020). Relocation not only generates additional costs, but it also restricts the access to partner's resources and sharing of costs.

Although divorcing spouses likely receive financial support from their parents, if parents are financially capable to help (Leopold & Schneider, 2011), inter vivos transfers in combination with divorcing individuals' personal incomes are unlikely to fully compensate all divorce‐related costs and prevent wealth declines. Indeed, previous research showed that marital dissolution is associated with a relatively abrupt and substantial decline in wealth levels compared to predivorce levels and that those declines are predominantly a result of declines in housing wealth (Kapelle & Baxter, 2021; Zagorsky, 2005). As continuously married individuals do not experience similar financial burdens, I expect that divorcees hold substantially less personal wealth in the year of divorce compared to otherwise similar, continuously married individuals (*Initial level hypothesis*).

### Long‐term development: Wealth accumulation after divorce

The unfavorable wealth position of divorcees right after divorce compared to the position of continuously married individuals may pose a relative disadvantage to divorcees. On average, more affluent married individuals would over‐proportionally benefit from exponential wealth growth over time based on compounded interest effects or asset appreciation. Thus, initial divorce‐induced wealth inequalities themselves become a detriment that theoretically lead to a systematic divergence of divorcees' wealth accumulation trajectories compared to continuously married individuals.

Furthermore, divorce may lead to restricted exposure to certain economic advantages, which could additionally inhibit divorcees' wealth accumulation over time. While continuously married couples benefit from marital wealth premiums including economies of scale or long‐term joint saving incentives (Lersch, 2017; Wilmoth & Koso, 2002), divorcees lack these benefits of first marriage. Also, divorcees may be bound to their ex‐spouse through financial ties (e.g., child and spousal alimony), which reduces divorcees surplus income that can be saved. Thus, I hypothesize that divorcees' yearly wealth accumulation rate is lower than the rate of continuously married respondents (*Growth rate hypothesis*), leading to a growing gap between divorcees and first‐time married spouses.

First, empirical support for the idea of a growing wealth divide between divorcees and the married was provided by Zagorsky (2005), who found that unpartnered divorcees had lower yearly saving rates compared to continuously married respondents—14% and 16%, respectively. However, considering only unmarried divorcees' wealth accumulation rates, his study relied on a selective sample of divorcees and neglected potential advantages associated with remarriage. Remarriage likely restores some marital advantages. For instance, remarried divorcees can benefit from improved economies of scale or tax benefits which may increase surplus income that can be saved. However, weaker beliefs about the longevity of higher‐order relationships and previous experiences of a divorce are linked to more financial independence within these higher‐order partnerships, which decreases the likelihood of—commonly more efficient—joint investments (Burgoyne & Morison, 1997). Thus, even remarried divorcees may not close the initial wealth gap generated as a result of their previous divorce. Nevertheless, it can be expected that divorcees' ability and motivation to save is moderated by remarriage with remarried divorcees experiencing higher wealth accumulation rates than unmarried divorcees (*Remarriage growth rate hypothesis*). As most divorcees eventually cohabit, but cohabitation has fewer wealth benefits, I focus on remarriage.

### Gender differences in the initial level effect and long‐term development

A growing body of research has highlighted substantial within‐marriage wealth inequalities to the disadvantage of wives (Grabka et al., 2015; Kapelle & Lersch, 2020). These inequalities stem, for instance, from a pervasive gender pay gap and women's lower access to wealth‐relevant fringe benefits, which are exacerbated during marriage due to persistently traditional arrangements of paid and unpaid labor (Chang, 2010). Additionally, research has highlighted age differences as well as gender differences in investment and spending as relevant contributors to the within‐marriage wealth gap (Alesina et al., 2013; Fisher, 2010). Finally, inter vivos transfers, which are legally not regulated, may also over‐proportionally favor husbands (i.e., sons) to endorse men's normative entitlement to relevant family‐of‐origin assets (e.g., property or businesses) (Bessière, 2019).

Depending on the origin of within‐couple wealth inequalities, divorce maintains or potentially exacerbates these inequalities. In the majority of Western societies including Germany, premarital wealth as well as personal inheritances and inter vivos received during the marriage commonly remain (largely) untouched in the divorce‐related equalization process meaning that inequalities in these wealth components are maintained. Although marital wealth (i.e., wealth accumulated during the marriage excluding personal inheritances or gifts) should de jure be divided equally or equitably—depending on the country—de facto arrangements may disadvantage women. Husbands are commonly perceived to be entitled to a larger share of marital wealth due to their, on average, higher economic contribution and an overall undervaluation of women's unpaid labor (Hersch & Shinall, 2020). These ideas have been found to be reflected in gender‐biased practices of family courts and divorce lawyers with endorsements of these practices by divorcees themselves across different country contexts (France: Bessière (2019); United States: Wenig (1990)). As a result of a potentially gender‐biased division of marital wealth, it can be anticipated that the initial wealth gap between men and women is larger for divorcees than the married (*Gendered initial level hypothesis*).

Gender is likely also a relevant moderator of wealth accumulation differences over time. Within marriage, wives' lower wealth accumulation potentials may to some degree—but not fully—be compensated as the majority of married spouses pool and share a substantial proportion of their resources (Amuedo‐Dorantes et al., 2011). However, after divorce, voluntary financial cooperation between ex‐spouses likely ceases and disparities in wealth accumulation potentials are no longer compensated. This is exacerbated by the fact that children commonly stay with their mother after divorce. Although postdivorce alimony and child support—if children are present—may cover some of the economic disadvantages, these payments are often considered insufficient with underpayment or nonpayment common issues (Skinner et al., 2017). Although remarriage can restore some of the economic advantages of marriage (Jansen et al., 2009), men are more likely to remarry and do so quicker than women (Coleman et al., 2000; Di Nallo, 2018). Overall, it can thus be expected that wealth accumulation disparities are more severe between divorced men and women than between married men and women (*Gendered growth rate hypothesis*).

## METHODOLOGICAL CHALLENGES

An analysis of how divorce potentially disrupts wealth trajectories in comparison to trajectories of continuously married individuals is methodologically challenging for two reasons. First, while divorcees have a date of divorce at which their divorce proceeding is completed and economic recovery can commence, continuously married naturally do not have such a comparison date. Thus, the question arises what point of time during the continuous marriage should be chosen as a comparison point for the year of divorce.

Second, initial wealth level differences between divorcees and the continuously married as well as differences in wealth accumulation rates between the two groups may substantially be determined by inherent differences between the two groups (i.e., selection effects). Previous research on the determinants of marital stability highlighted a range of predictive characteristics including, inter alia, the prevalence of financial issues, spouses' socioeconomic background including parental separation, or ownership and level of specific assets or liabilities (Amato, 2010; Dew, 2011; Eads & Tach, 2016).

To address these two challenges, I used a combination of propensity score and coarsened exact matching (see van Scheppingen and Leopold (2020) for a similar methodological approach). To this end, I systematically selected continuously married survey respondents that were most alike to married respondents that eventually experienced a divorce during their panel participation. In this process, continuously married control respondents could be assigned the date of divorce of their treated match (i.e., divorcees). This means that a common time scale of *time since (assigned) divorce* could be generated for the treatment and control group. Second, the matching addressed a wide range of inherent differences between the two groups and thus reduced issues of selectivity.

## THE GERMAN CONTEXT

Given that this study draws on German data, it is important to understand the specific context that may influence wealth stratification processes relevant for the present study.

### Economic inequalities

Although formal measures of economic wellbeing and security have placed Germany on average on a secure footing, economic inequalities have been soaring in recent decades with Germany ranking amongst the EU countries with the highest wealth inequalities (European Central Bank, 2020; Piketty, 2014). At the same time, households' and individuals' ability to accumulate sufficient wealth has become a critical issue as Germany's government has increasingly emphasized personal responsibility to ensure reasonable living standards throughout the life course amid an aging population and rising economic pressure on the government (Ebbinghaus, 2015).

### Marriage premium

Wealth accumulation in Germany is strongly linked to marriage. Strong normative expectations around joint savings within marriage are endorsed through institutional structures and privileges for the married compared to nonmarried (i.e., singles or cohabiters). Married spouses, but not cohabiters, can financially benefit from favorable taxation or joint insurances and pensions (Bach et al., 2013). Additionally, access to the housing market is largely restricted to the married and homeownership is often seen as a once‐in‐a‐lifetime opportunity as property acquisition in the prudential German mortgage system requires substantial deposits and income security (Thomas & Mulder, 2016).

### Economic gender differences

Structural privileges for the married rest on the notion of traditional gender roles and specialization within marriage. This has provided strong incentives for German wives to reduce their work hours and resulted in comparatively low rates of full‐time employment amongst women (Aisenbrey & Fasang, 2017; Trappe et al., 2015). In combination with occupational segregation and undervaluing of jobs within female‐dominated industries and occupations, traditional family arrangements have carried a significant penalty for German wives' relative earnings and wealth (Grabka et al., 2015; Trappe & Sørensen, 2006).

### Divorce the German way

Germany's divorce rate per 100 marriages (2019: 36) is comparable to the US rate (2019: 37) (CDC, 2021; Statistisches Bundesamt, 2021). On average, German women and men are aged 44 and 47 respectively at divorce, and divorce takes place after 14.8 years of marriage (Statistisches Bundesamt, 2021). To legally divorce, Germany requires spouses to live separately for at least 12 months prior to divorce (United States: 6–12 months depending on the state). At divorce, the default German matrimonial property regime emphasizes a de jure equal division of marital property (i.e., assets and liabilities accrued during the marriage excluding personal inheritances or gifts) through an equalization of accrued gains. This means that the wealthier spouse is required to make an equalization payment to the less wealthy spouse amounting to half the difference in accrued gains. The divorce proceeding itself incurs substantial, although capped and regulated, administrative cost (i.e., court fees and solicitors' fees) that increase with the complexity of the case and the level of financial value of goods and property in dispute.

The strong institutional support for women's economic reliance on husbands during marriage stands in contrast to Germany's legal emphasis on independence between spouses after divorce. For instance, the division of marital property does not consider the future need of the economically less advantaged spouse (i.e., commonly the wife) as is common in some US states (Voena, 2015). Furthermore, postdivorce spousal alimony regulations, which were tightened in 2008, emphasize the principle of financial self‐sufficiency with alimony temporarily limited—if granted at all. For divorces that involve dependent children, monetary child support must be paid by the nonresidential parent—commonly the father. Nevertheless, only a minority of residential parents receive child support from their ex‐partner and only half of all payments are sufficient (Bröckel & Andreß, 2015). This is particularly detrimental to women since the majority of children stay with mothers after divorce. According to Walper (2018), 84% of children reside with mothers after marital dissolution while only 7% reside with the father; 9% of children live in shared residential arrangements. Overall, German women have been found to experience high and lasting financial volatility after divorce compared to German men partially due to inequalities generated during marriage. German women also fare worse compared to women in other countries such as the United States (Bayaz‐Ozturk et al., 2018).

## DATA AND METHODS

### Data

I used longitudinal panel data from the German SOEP (v36; Goebel et al. (2019)). The SOEP is a representative panel study of German households that commenced in 1984. The data are well suited for the analysis of wealth trajectories of divorcees and continuously married individuals including an approach that considers remarriage and gender, as they contain (a) retrospective marital biographies that are updated yearly with prospective data, (b) comprehensive measures of *personal* wealth in four survey waves (2002, 2007, 2012, 2017), and (c) a wide range of other relevant annually measured covariates. Whereas the outcome regression only used data from the four wealth waves (i.e., 2002, 2007, 2012, 2017), I relied on yearly survey data for years 1984–2017 for the matching.

I used wealth data that were edited and imputed by the SOEP survey team (Grabka & Westermeier, 2015). I additionally multiply imputed other analytical variables and auxiliary variables using Stata's *mi* command resulting in five imputation sets. Estimation results from each imputed set of data were combined using Rubin's rule (Rubin, 1987).

### Sample selection and matching

**FIGURE 1:**
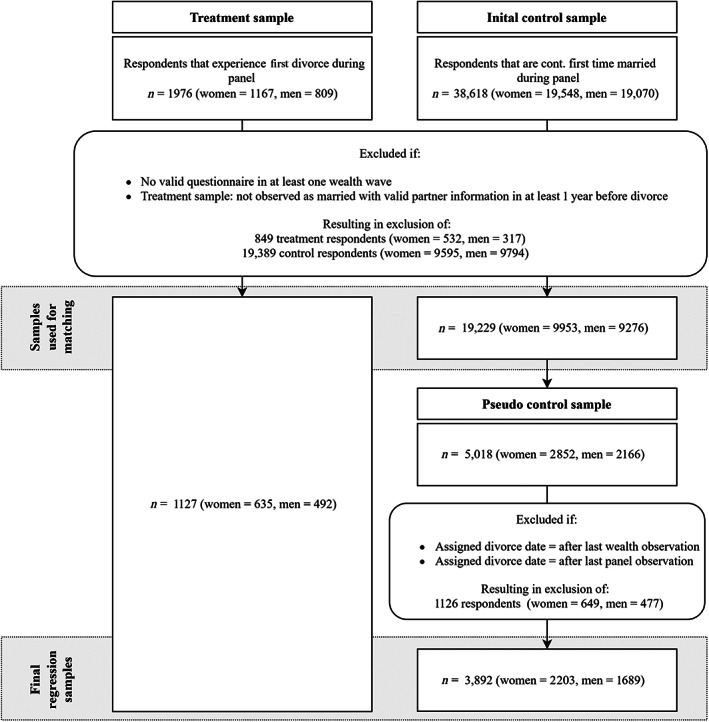
Overview diagram: sample selection and generation process

The following section will elaborate on the sample selection and generation process including the generation of a pseudo control sample through matching. Figure 1 provides a graphical representation of the rather complex process.

#### Initial sample selection

In the first step of the sample selection process, I generated two samples: the treatment sample and the preliminary control sample. For the treatment sample, respondents were selected if they experienced a divorce from their first marriage during panel participation, were observed as being married in at least one survey year before their divorce with valid partner information in this year, and provided a valid questionnaire in at least one wealth survey year after their divorce. The preliminary control sample included respondents that had been observed as continuously first‐time married during their panel participation and provided valid information in at least one wealth survey year. To connect the present study to the previously discussed studies that focused on wealth of ever‐divorced individuals in late working age and reduce the influence of wealth‐declines associated with retirement, individuals were no longer followed once they were aged 61 years. Additionally, respondents were no longer followed once they experienced the death of their spouse. These first sample selection criteria resulted in a treatment sample of 1127 individuals (635 women and 492 men) and a preliminary control sample of 19,229 individuals (9953 women and 9276 men).

#### Generation of a pseudo control group

The second step of the sample selection process involved the generation of a pseudo control group using matching. This means that from the initial 19,229 continuously married respondents those respondents were selected that resembled treatment group respondents on 36 relevant predivorce characteristics using nearest neighbor propensity score matching in combination with (coarsened) exact matching. Matching variables covered inter alia respondents' basic demographics, family of origin characteristics, household living arrangements and health status of members, or the financial situation of respondents and their partners. For the matching, only variables that were measured on an annual basis could be considered. Although wealth values would have ideally been included in the matching process, they were not measured on a yearly basis. To address this shortcoming and cover relevant aspects of the wealth portfolio and wealth accumulation potential, I included covariates for respondents' and their partners' personal income, education, and subjective economic wellbeing as well as household‐level categorical measures of the wealth portfolio (e.g., homeowner, shares, capital gains, etc.). Respondents were matched in the earliest available year of their marriage in which respondents and their partners had valid interviews. Thus, for respondents that entered their marriage during the panel participation, the matching year was likely one of the first years of their marriage. For respondents that entered the panel as being married, the matching year was likely one of their first panel participation years. The decision to match on the earliest available year was necessary to ensure that even short marriages could be considered in the current study and to reduce the influences of anticipation effects (i.e., spouses may change their financial behavior in the years prior to their marital dissolution). A more detailed, technical description of the matching process including an overview of considered variables is provided in the Supporting Information. Each divorcee was matched to up to five best matches. As common for similarly sized datasets, the matching was conducted with replacement, meaning that respondents in the control sample were included more than once. This guaranteed that each divorcee could be matched to the most appropriate nearest controls, even if these control respondents were already included in a previous match (Dehejia & Wahba, 2002). In total, the 1127 divorcees were matched to 5018 continuously married control sample individuals.

#### Assignment of divorce dates to pseudo control group respondents and final regression sample restrictions

For the subsequent growth models, respondents in the pseudo control group were assigned the divorce date of their treatment matches. Years prior to (assigned) divorce were dropped, as the subsequent outcome analysis focused on wealth trajectories after divorce. Additionally, the matched sample had to be restricted to survey years that contained wealth information. In some cases, the assigned divorce date was after the last wealth observation or the last valid panel observation, which resulted in a loss of 1126 respondents in the pseudo control group. Additional analyses confirmed that dropped respondents did not substantially differ from control respondents that remained in the sample. Overall, the regression sample consisted of the initial 1127 divorcees (635 women and 492 men) with 2067 individual‐year observations, and a pseudo control group with 3892 (2203 women and 1689 men) respondents with 7734 individual‐year observations. The regression sample was unbalanced, with an average of 2.0 person‐years per respondent. Table S3 provides descriptions of the samples.

### Outcome regression measurements

#### Outcome variable

My outcome measure, *personal net wealth*, was defined as the sum of all personally owned assets minus personally owned liabilities including personal share in jointly held assets and liabilities. Assets covered real estate, financial assets (e.g., savings balance, shares, or bonds), life insurance, private pension plans, business assets, and valuable assets (e.g., gold, jewelry). Liabilities covered mortgage debt and consumer credits. SOEP wealth data are currently available for the years 2002, 2007, 2012, and 2017, and have been collected separately for each household member aged 17 and older in a three‐step process: (1) a filter question is used to assess whether a respondent holds a certain wealth component; (2) the market value of held wealth components is recorded; and (3) for wealth components that may be held jointly (e.g., real estate), respondents are asked to indicate whether they hold these wealth components solely or jointly and—in the case of joint ownership—provide the share they co‐owned. My outcome measure thus explicitly included the personal share of any assets and liabilities that were owned by other individuals. I adjusted personal net wealth for inflation using the consumer price index and top‐ and bottom‐coded the extreme 0.1% of reported wealth measures.

Although wealth data are commonly skewed and transformations are applied, analyses for the current study were conducted with untransformed data. This was necessary because transformations of wealth hamper the accessibility of results for nonwealth researchers and would make interpretations of growth rates particularly inaccessible. For example, divorcees and the married may both experience an average yearly wealth increase of 5%, but if divorcees have overall lower wealth levels than the married, similar yearly increases in relative wealth translate into drastically lower absolute increases for divorcees compared to the married. To assess the general robustness of results and particularly initial level differences amid data skewness, main analyses were repeated using an inverse‐hyperbolic sine transformation and rank‐based transformation (e.g., Friedline et al., 2015; Killewald et al., 2017). Overall, these robustness analyses confirmed the main results.

Previous research showed that housing wealth is more likely accumulated jointly than other wealth components during marriage (Joseph & Rowlingson, 2012). In addition, the marriage wealth premium was found to be more pronounced for housing wealth particularly for women, while marriage also increases men's financial wealth (Kapelle & Lersch, 2020; Lersch, 2017). In the case of divorce, research found strong housing wealth declines for both men and women whereas financial wealth declines were more moderate particularly for men (Kapelle & Baxter, 2021). Therefore, I disaggregated wealth into housing wealth and financial wealth for additional analyses.

#### Explanatory variables

To model wealth growth trajectories over time after divorce, I first generated a continuous variable to measure *time since (assigned) divorce*. This variable started with 0, representing the year of divorce, and increased by 1 for each year since first divorce. For the pseudo control group, this variable represented an artificial count since their assigned divorce date. Time since divorce covered up to 30 years, although the sample size was reduced during later years after divorce. For the regression analyses, time since divorce was included as a linear term. To distinguish and assess wealth trajectory differences between the control and treatment group, I generated a dummy variable to tag respondents with and without an actual *divorce experience* (0 = control [ref.], 1 = treated). To examine whether *remarriage* moderates wealth trajectories of divorcees, I used a dummy variable (0 = not observed as remarried [ref.], 1 = remarried). Finally, for the assessment of potential gender differences, I generated a *gender* dummy (0 = male [ref.], 1 = female).

#### Control variables

The multivariable regression models were controlled for a small set of time‐changing covariates. To account for potential underreporting of personal wealth in the first observed wealth wave, I included a flag for respondents' first wealth observation. Additionally, I flagged imputed wealth data using a dummy variable. To account for changes in the German spousal alimony law in 2008 and the economic crises (2007–2009), I included a dummy to tag years prior to wealth waves 2012 and 2017. Finally, wealth differences between Eastern and Western Germany were accounted for by a dummy that indicated whether a respondent was currently living in Eastern Germany or not. Other covariates were not included because baseline differences between the treatment and control groups were adjusted for in the matching approach.

### Multivariable random‐effects growth model

I used random‐effects growth models with random intercept and random slope to predict initial‐level personal wealth and yearly personal wealth increases over time for divorcees and the continuously married (Singer & Willett, 2003). These models were most suitable as they can deal with the nested structure of the data but can also handle unbalanced data and unequal spacing or numbering of measurements across respondents.

I commenced the analysis by specifying the following general model:

$${\text{WEALTH}}_{\mathrm{it}}=\left[\gamma_{00}+\gamma_{10}{\text{DIVTIME}}_{\mathrm{it}}+\gamma_{01}{\mathrm{DIV}}_{i}+\gamma_{11}\left({\mathrm{DIV}}_{i}*{\text{DIVTIME}}_{\mathrm{it}}\right)+\gamma_{0k}C_{\mathrm{it}}\;\right]+\left[\zeta_{0i}+\zeta_{1i}\mathrm{DIV}{\text{TIME}}_{\mathrm{it}}+\varepsilon_{\mathrm{it}}\right].$$

The first parenthesis contains the structural component of the model, while the stochastic component is represented within the second parenthesis. ${\text{WEALTH}}_{\mathrm{it}}$ is the personal wealth of respondent i at time t. The average intercept is captured by $\gamma_{00}$ with the random component $\zeta_{0i}$. The random component represents individual‐specific variation in the intercept that is unexplained due to unobserved characteristics of individuals. ${\text{DIVTIME}}_{\mathrm{it}}$ represents the years since (assigned) divorce. The related average growth slope over time is denoted by $\gamma_{10}$, which may vary across individuals and is captured by $\zeta_{1i}$. I allowed the random components, $\zeta_{0i}$ and $\zeta_{1i}$, to be correlated. This means that time‐constant respondents' characteristics could simultaneously modify the intercept (i.e., initial level) and slope (i.e., growth rate) of personal wealth. I further included a dummy, DIV, that identifies whether respondents belong to the pseudo control group (i.e., continuously married) or the treatment group (i.e., divorced) with the corresponding coefficient $\gamma_{01}$. This means that the term $\gamma_{00}$ relates to the average intercept of the control group, whereas $\gamma_{01}$ describes the treatment group's variation from the average intercept providing an indication for my *Initial level hypothesis*. Additionally, I included an interaction between years since divorce and the treatment dummy, ${\mathrm{DIV}}_{i}*{\text{DIVTIME}}_{\mathrm{it}}$, with the corresponding coefficient $\gamma_{11}$; this subsequently relates to the treatment group's slope variation from the control group's intercept, $\gamma_{10}$. The inclusion of the interaction thus allowed me to test the *Growth rate hypothesis* (i.e., yearly wealth increases). Finally, $C_{\mathrm{it}}$ is the set of *k* control variables.

In the next step, I tested whether remarriage moderates growth rates (*Remarriage growth rate hypothesis*). To this end, I included a three‐way interaction between time since divorce, the divorce dummy, and an indicator for ever remarried after divorce into my initial model. Note that continuously married respondents are naturally never remarried and thus some of the interaction predictions fell out of the model.

For the final model, I included a three‐way interaction between time since divorce, the divorce dummy, and gender into my initial model to address how gender moderates wealth trajectories of divorcees and the married. Results of this model in combination with Wald tests enabled an empirical assessment of the *Gendered initial level hypothesis* and *Gendered growth rate hypothesis*.

## RESULTS

**FIGURE 2:**
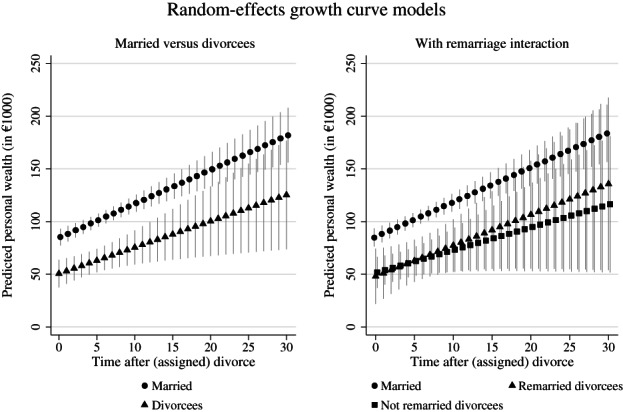
Random‐effects growth models: predicted personal wealth trajectories for divorcees and continuously married overall and including an interaction for remarriage. *Note*: Whiskers indicate 95% confidence intervals. Data are from the Socio‐Economic Panel Survey v36 (2002, 2007, 2012, 2017; unweighted; multiply imputed). Full model results are provided in Table S2

**FIGURE 3:**
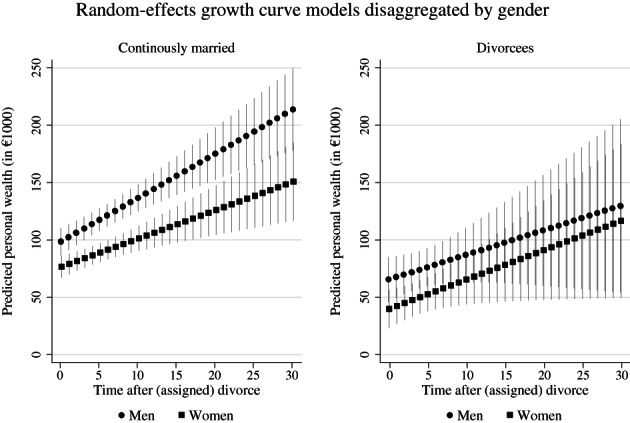
Random‐effects growth model: predicted personal wealth trajectories for divorced and continuously married men and women. *Note*: Whiskers indicate 95% confidence intervals. Data are from the Socio‐Economic Panel Survey v36 (2002, 2007, 2012, 2017; unweighted; multiply imputed). Full model results are provided in Table S2

Regression results are displayed graphically in Figures 2 and 3. Detailed regression results are provided in Table S2.

### Initial wealth disparities and differences in yearly wealth accumulation rates

First, I hypothesized that in the year of divorce, divorcees hold substantially less personal wealth than otherwise comparable, continuously married individuals due to divorce‐related wealth depletions (*Initial level hypothesis*). In line with these expectations, I found that divorcees held €34,661 less wealth in the year of divorce compared to married respondents. Adjusted for covariates included in the regression, married respondents had an average predicted initial personal wealth level of €85,243. The difference between wealth levels of the married and divorced at the time of (assigned) divorce was substantial and statistically significant.

Furthermore, I expected that divorcees accumulate wealth at lower yearly rates compared to their continuously married counterparts due to divorcees' restricted access to wealth‐accumulation‐related benefits (*Growth rate hypothesis*). Lower yearly wealth increases amongst divorcees would lead to a growing gap between the married and divorced over time. As visible in Figure 2, yearly wealth increases were only marginally different between the two groups in contrast to theoretical expectations. Whereas the married increased their personal wealth by €3225 per year, which was a statistically significant increase, divorcees' average yearly personal wealth increase of €2488 (€3225 − €737) was marginally below married respondents. The difference of €737 was statistically not significant.

However, the first model considered divorcees as a homogeneous group although divorcees likely differ in their wealth accumulation depending on whether they remarry or stay unmarried (*Remarriage growth rate hypothesis*). Including an interaction between time since divorce, the divorce dummy, and an indicator for ever remarried after divorce into the regression model highlighted that remarried divorcees indeed showed substantially higher yearly personal wealth increases after divorce than unmarried divorcees. Precisely, remarried divorcees increased their wealth on average by €2921 (€3232 − €1077 + €766) per year, which was similar to increases of continuously married respondents, whereas unmarried divorcees experienced yearly average increases of only €2155 (€3232 − €1077). Thus, particularly unmarried divorcees increased their initial gap to the married over time. Although growth rates between the three groups did not differ statistically, effect sizes were in the expected direction supporting the idea that remarriage moderates divorcees' wealth growth rates (*Remarriage growth rate hypothesis*).

### Gender‐specific effects in initial wealth disparities and yearly wealth accumulation rates

Based on notions around the gender wealth gap and gender‐biased practices during the marriage and in the case of divorce, it was critical to explore potentially relevant gender differences in wealth trajectories of divorcees and the married.

I argued that divorce‐related wealth declines would be more considerable for women than men. Thus, the initial wealth gap between men and women could be expected to be larger for divorcees than for the married (*Gendered initial level hypothesis*). Overall, I found substantial and significant gender gaps for both divorcees and the married. Whereas married women held €21,808 less personal wealth in the year of assigned divorce compared to men, who had an average personal wealth level of €98,425 in this year, divorced women held on average €25,625 (€21,808 + €3817) less personal wealth than divorced men, who had an average predicted personal wealth level of €65,479 in the year of divorce. As suggested in the *Gendered initial level hypothesis*, I found a gender gap in initial personal wealth levels that was almost €3817 higher for divorcees than the married. This translated into an 18% higher gap for divorcees than the married. Although this difference was statistically not significant according to a Wald test, it can be considered substantial.

Moreover, I expected to find larger gender disparities in wealth accumulation rates for divorcees than for the married due to a compensation of women's lower wealth accumulation potentials within marriage and a lack of such compensation after divorce (*Gendered growth rate hypothesis*). Thus, initial gender differences should widen more for divorcees than the married. Regression results confirmed that, on average, married women accumulated wealth at considerably lower rates than married men, €2473 (€3842 − €1369) and €3842 respectively. However, contrary to theoretical expectations of the *Gendered growth rate hypothesis*, results indicated that divorced women accumulate wealth at marginally higher rates than divorced men, €2541 (€3842 − €1369 − €1701 + €1769) and €2141 (€3842 − €1701) respectively. The yearly differences of €400 to the advantage of divorced women over men was, however, statistically not significant. Nevertheless, I conducted additional analyses to further explore whether results could be explained by employment and family characteristics (results are available from the author upon request). Including measures for employment status, personal labor market income, partnership status, and number of children partially explained divorced women's marginally higher wealth accumulation rate compared to divorced men's rate. In the adjusted model, divorced women increased wealth on average by €2024 whereas divorced men's increase was marginally higher at €2419.

### Wealth trajectories in housing and financial wealth

To further explore underlying patterns that may explain some of the results, I additionally examined housing wealth and financial wealth separately (see Figures S3–S6). Disaggregated results showed that divorcees held substantially less housing wealth in the year of divorce while differences in financial wealth were marginal, in line with previous research (Kapelle & Baxter, 2021). Over time, divorcees accumulated housing and financial wealth at similar rates to the married. However, remarriage importantly moderated the postdivorce accumulation of housing wealth, but not financial wealth. Precisely, remarried divorcees accumulated housing wealth at higher rates than the continuously married and unmarried divorcees although accumulation rates were insufficient to close the gap to the married even after 30 years.

Financial wealth was particularly relevant to explain gender differences in wealth trajectories between married men and women. Housing wealth trajectories of the married followed parallel trends, in line with previous research (Joseph & Rowlingson, 2012; Kapelle & Lersch, 2020). Thus, sizeable gender differences in initial wealth levels and yearly wealth increases between continuously married men and women were predominantly due to women's substantially lower accumulation of financial wealth. Not surprisingly, results further illustrated that initial gender differences at divorce were almost exclusively due to differences in financial wealth. These differences in financial wealth were reduced over time as divorced women accumulated financial wealth on average at slightly higher rates than divorced men. However, divorced men accumulated housing wealth at higher rates over time, which generated small housing wealth disparities between divorced men and women in years after divorce.

### Robustness analyses

To assess the robustness of the presented results, I conducted a range of additional analyses. First, I addressed issues of reduced cell sizes in the duration since (assigned) divorce in later years. Cell sizes were particularly reduced after 15–20 years after divorce (see Table S4). As single outliers in later years with only a few sample respondents in those years can influence regression results, I estimated regression analyses first by excluding postdivorce years larger than 20 and second excluding years larger than 15 years from the analyses. While this led to a reduction in the sample size, results of this supplementary analysis were consistent with the main results (see Figures S6 and S7).

Second, I included a quadratic term for time since (assigned) divorce because wealth accumulation commonly follows an inverse u‐shaped pattern over the life course. The inclusion of a quadratic term did not improve the model fit or change results. This was likely because retirement‐related wealth accumulation changes were not captured in the present study due to the focus until late working age.

Third, although the imputed SOEP wealth data compare well to wealth data based on the economic balance sheets of the German Federal Statistical Office and Deutsche Bundesbank (Grabka & Westermeier, 2015), I estimated regression analyses without imputed data. Results were overall consistent and substantial interpretations remained the same.

Finally, I addressed the potential issue of sample attrition. Although I found no evidence of selective attrition by wealth following divorce, I nonetheless estimated my models using longitudinal weights that accounted for attrition. Results from weighted regressions were consistent with those presented above. I thus present the more parsimonious models in this manuscript. Results for all robustness analyses are available from the author upon request.

## DISCUSSION AND CONCLUSION

Against the scenario of historically high divorce rates and a rising relevance of access to sufficient private wealth during a time of soaring wealth inequalities, an incipient body of previous research highlighted substantial wealth disparities between preretirement ever‐divorced and continuously married respondents (e.g., Wilmoth & Koso, 2002; Zissimopoulos et al., 2015). Using a cross‐sectional approach and household‐level wealth data, research had left the question of how divorce is linked to the stratification of wealth unaddressed. The present study filled this gap by theoretically and empirically scrutinizing wealth disparities between ever‐divorced individuals and continuously married individuals as a result of immediate wealth declines associated with divorce and potentially deteriorated wealth accumulation potentials of divorcees compared to the married after divorce. Furthermore, I expected remarriage and gender to importantly moderate wealth trajectory differences. To test my expectations, I used longitudinal data from the German SOEP and applied a novel doubly robust estimation approach that combined propensity score and (coarsened) exact matching with an outcome regression to provide more robust estimates of wealth trajectories of divorced and married respondents while accounting for selection into divorce.

My study presents several original and relevant findings. First and foremost, results illustrated that in line with notions of my *Initial level hypothesis* initial wealth differences generated around divorce are likely the main driver of lasting wealth disadvantages for ever‐divorced compared to continuously married individuals rather than differences in wealth accumulation rates over time. Initial differences were particularly pronounced for housing wealth rather than financial wealth. This is in line with previous research that illustrated high probability of homeownership loss at divorce (Lersch & Vidal, 2014). Although it may be expected that property sales and thus declines in housing wealth result in increasing financial wealth, this is not necessarily the case in Germany where housing sales also incur substantial costs (e.g., speculation taxes, mortgage early repayment charges, transfer taxes, etc.).

In contrast with my *Growth rate hypothesis*, differences in yearly wealth accumulation rates were statistically not significant and only marginal although effects were in the expected direction. Disaggregating wealth into housing and financial wealth did not show relevant differences in wealth accumulation rates between divorcees and the married either. While the lack of a wealth accumulation scarring effect is surprising, several explanations can be discussed. First, to benefit from compounded interest effects, individuals need to have a substantial amount of wealth invested in assets that yield returns of investments (e.g., shares). This may not be the case for the majority of married or divorced individuals. Thus, it is possible that the “average” divorced and married individuals may not have substantially different access to the benefits of compounded interest effects. Due to data limitation, a detailed exploration of this possible explanation was not feasible. Second, continuously married individuals were more likely to hold personal wealth in housing wealth, which has often been associated with wealth‐building advantages. Nevertheless, Lersch and Dewilde (2018) showed that although Germans increase their financial wealth substantially leading up to the entry into homeownership (i.e., goal‐oriented saving), once they are homeowners they reduce their probability to save and the rate at which they save. Thus, higher homeownership amongst the married is not necessarily associated with higher saving rates as also illustrated by the parallel wealth trajectories found for financial wealth. Finally, while divorcees accumulate wealth at similar rates as the married at the average, this average effect may obscure substantial underlying heterogeneity. Indeed, I found that remarriage moderates wealth growth rates of divorcees in line with my *Remarriage growth rate hypothesis*. This was particularly a result of remarried divorcees' higher housing wealth accumulation compared to never‐remarried divorcees. This is not surprising in the German context where down payments, transaction costs, and monthly repayments may often be out of reach for singles and are exceptionally high in the international comparison (Thomas & Mulder, 2016).

Although divorce‐related wealth declines can certainly not be completely avoided, my results indicate that discussions on how divorcees can be supported in their economic self‐reliance should focus on how immediate costs associated with divorce may be reduced or/and how divorcees could achieve saving rates that outperform the married—although the latter seems particularly unfeasible. As initial level differences were found to be predominantly a result of divorcees' lower housing wealth in the year of divorce, mitigation approaches may focus on how homeownership can be maintained or reentry into homeownership eased after divorce. This seems particularly critical in the German context where the prudential mortgage system and high transition costs have created a segregated homeownership market that is largely restricted to married couples and discourages reentry into the market. Policy discussions may focus on how Germany's homeownership market could be made more accessible but nevertheless stable (e.g., lower transaction costs, rent‐to‐own schemes, etc.).

Furthermore, my study highlighted substantial gender differences in wealth accumulation trajectories. Both married and divorced women were found to hold less personal wealth than men in the corresponding groups at the time of (assigned) divorce. Initial gaps were slightly larger for divorcees in line with my *Gendered initial level hypothesis* although the difference in the gender gaps was statistically not significant. For married spouses, this result supports previously found gender wealth gaps within marriage (Grabka et al., 2015; Kapelle & Lersch, 2020). For divorcees, results highlight that the common assumption of an equal division of *all* available resources at divorce is unlikely to hold as also suggested by Kapelle and Baxter (2021). This is particularly the case for financial wealth, which was found to drive gender differences in wealth levels at divorce. However, it needs to be acknowledged that previous research—which assumed equality in the division of all household wealth due to data restrictions (e.g., Zagorsky, 2005)—referred to the US context. In the United States, an equal division of wealth is often considered desirable and future needs of spouses are regularly considered in the property division. In this context, judges have more discretion in divorce cases than in any other field of private law. This may indeed lead to lower gender inequalities in postdivorce wealth in the United States than in Germany. Thus, for future research, it will be relevant to consider the association between divorce and wealth in different contexts to understand how different legal frameworks and policies matter.

Finally, the results did not support my *Gendered growth rate hypothesis*. Rather results indicated that wealth accumulation differences were substantially larger between married men and women, while differences were negligible between divorced men and women. Disaggregating net wealth, it became clear that differences between married men and women were exclusively driven by men's substantially higher yearly accumulation of financial wealth while housing wealth was accumulated at similar rates. This is in line with previous research that highlighted that housing wealth is commonly owned jointly and thus accumulated equally within marriage (Joseph & Rowlingson, 2012; Kapelle & Lersch, 2020). The low differences in yearly wealth accumulation rates between divorced men and women could not be explained by differences in employment or family characteristics. Results further indicated that while divorce men had marginally higher accumulation rates of housing wealth, divorced women outperformed men in the accumulation of financial wealth. Results for divorcees may indicate that women are aware of their financial vulnerability and change their financial behavior after divorce to defeat their lower wealth accumulation potentials. Alternatively, it is also likely that financially more independent women are more likely to experience a divorce while financially less successful men are particularly likely to get divorced. Indeed, previous research commonly found that men's unemployment generally has a higher predictive power of divorce than wives' unemployment (Jalovaara, 2003; Killewald, 2016). At the same time, women's rising educational and economic achievements have been discussed as a divorce destabilizer by some (Raley & Sweeney, 2020).

Overall, results highlight that gender differences that were previously found between marital groups in older age likely have a complex origin, and addressing those differences requires multifaceted approaches. This emphasizes the importance to improve gender equality throughout the life course rather than solely focused on time after divorce.

Two limitations of the present study are noteworthy. First, although the SOEP data are exceptional in the way they measure wealth longitudinally and at the personal level, the statistical analyses of the present study were restricted by the limited number of waves currently available. This resulted in a limited number of repeated observations and a limited timespan each respondent could contribute to the synthetic growth curves. Although the applied methods can deal with unbalanced panels, predictions may have been more stable with a higher number of reoccurring observations. Additionally, it cannot be ruled out that analyses could be biased by potentially systematic differences between cohorts. The limited number of wealth waves also meant that predivorce wealth trajectories could not be considered in the analyses or the matching. For the matching, I relied on a wide range of wealth proxies instead. Finally, it should be noted that the limited number of waves may have threatened causal inferences that can be drawn from the current study. Second, my analyses share a further limitation with other studies: the reliance on self‐reported personal wealth. While the collection of survey‐based wealth data requires a high level of financial awareness and knowledge on the part of respondents, the collection of personal wealth within the SOEP additionally requires respondents to make a judgment about their share of jointly owned assets. Nevertheless, it needs to be acknowledged that the data are unique in their provision of fully disaggregated wealth. As the access to individual‐level administrative wealth data is limited, the SOEP remains the most reliable source of comprehensive, longitudinal personal‐level wealth.

Overall, this study provides both a theoretical and empirical understanding of how divorce is linked to wealth stratification and contributes to soaring wealth inequalities. Precisely, the study was the first to thoroughly consider wealth disparities between the married and ever‐divorced as a result of (1) divorce‐related immediate wealth penalties and (2) potentially long‐term wealth accumulation disparities. Although divorce‐related wealth declines can certainly not be completely avoided, knowledge about the timing of wealth declines is critical for policymakers and practitioners alike to commence a discussion about whether and how interventions (e.g., subsidized legal aid, capped court costs, policies that provide a more inclusive homeownership market) could minimize wealth‐repercussions associated with divorce and improve divorcees' capabilities of economic self‐sufficiency throughout their life course.

## Supporting information


**Appendix S1**. Supporting Information.
