# Supplementary material for: Time cannot heal all wounds: Wealth trajectories of divorcees and the married
Source: J Marriage Fam. 2022 Jan 29;84(2):592–611. doi: 10.1111/jomf.12824 (PMC9303434; doi:10.1111/jomf.12824)
Supplement: Supplementary file 1 — Appendix S1. Supporting Information. [file JOMF-84-592-s001.docx]

Supplementary Material

# Propensity Score and Coarsened Exact Matching

To select continuously married respondents that were most alike to respondents that eventually experience a divorce during their panel participation, I used nearest neighbor propensity score matching in combination with exact matching. To this end, I first predicted the likelihood to experience a divorce (i.e., propensity score) by regressing 33 covariates on the binary treatment variable ‘divorce’ (see Table S.1 for the full list of covariates). In the second step of the matching process, I matched each divorcee to up to five best matches (i.e., nearest neighbor algorithm) of the control group based on the logit of the propensity score using calipers of width equal to 0.2 of the standard deviation of the logit of the propensity score. In addition to the propensity score, I also matched on three variables that seemed particularly relevant for the current study: the year of marriage, age, and gender. To ensure sufficient cell sizes, exact matching was only feasible for gender and the year of marriage whereas age had to be (partially) coarsened. Matching was conducted with replacement, meaning that respondents in the control sample were included more than once. This guaranteed that each divorcee could be matched to the most appropriate nearest controls, even if these control respondents were already included in a previous match. As multiple imputed data were used, I averaged the *m* propensity scores for each respondent across the completed datasets, then performed the matching with these averaged scores.

To provide a numerical indication of the quality of the matching, I calculated the standardized mean differences (Cohens’ *d*) between the treatment and control group before the matching and after the matching for all variables used in the generation of the propensity score. A graphical representation of standardized mean differences can be found in Figure S.1. Additionally, Table S.2 provides detailed information on means and standard deviations of covariates measured in the matching year of the divorce sample and the control sample before and after the matching. Standardized differences ranged between .00 and .74 before the matching. After the nearest neighbor propensity score and (coarsened) exact matching, the standardized differences ranged between .00 and .10, indicating that the balance of observable characteristics was substantially improved.

**Table S.1** List of covariates used for propensity score and (coarsened) exact matching

| **Basic demographics** | - Female [yes/no]^+^ - Age [continuous for propensity score, categorical for coarsened exact matching]^+^ - Cohorts [<1946, 1946-1955, 1956-1965, 1966-1975, >1976] - Migration background [yes/no] |
| --- | --- |
| **Family of origin** | - Number of siblings [continuous] - Parental education [low, intermediate, high] |
| **Marital status** | - Year of marriage [categorical]^+^ |
| **Living arrangements** | - Number of household members aged 0 to 17 years [continuous]^*^ - Number of household members aged 18 years and over [continuous]^*^ - Currently living in Eastern German federal state [yes/no]^*^ |
| **Health status** | - Someone in the household needs care/assistance on a constant basis due to age, sickness, or medical treatment [yes/no]^*^ |
| **Human capital and financial situation** | - Educational achievement [low, intermediate, high] - Personal earnings (log) [continuous] - Equalized household post-government income (log) [continuous]^*^ - Employment status [full-time, part-time, not in employment] - Full-time labor market experience since entry into labor market [continuous] - Satisfaction with household income [10 point Likert scale] - Worries about own financial situation [very concerned, somewhat concerned, no financial concerns] |
| **Wealth** | - Homeownership [yes/no]^*^ - Savings account ownership [yes/no]^*^ - Ownership of business assets [yes/no]^*^ - Holding building loan [yes/no]^*^ - Life insurance [yes/no]^*^ - Ownership of shares [yes/no]^*^ - Capital gains [none, under 250 Euro, 250 to under 1000 Euro, 1000 and more]^*^ |
| **Partner-level characteristics** | - Age [continuous] - Migration background [yes/no] - Number of siblings [continuous] - Parental education [low, intermediate, high] - Educational achievement [low, intermediate, high] - Employment status [full-time, part-time, not in employment] - Full-time labor market experience since entry into labor market [continuous] - Satisfaction with household income [10 point Likert scale] - Worries about own financial situation [very concerned, somewhat concerned, no financial concerns] |

*Notes:* *Variables measured at the household level. ^+^ Variables used for (coarsened) exact matching

Additionally, a graphical representation of the overlap of the propensity score provided an indication on how well the control sample matched the treatment sample in the distribution of the propensity score before and after the matching. As can be seen in Figure S.2, the matching substantially improved the overlap of the distribution of the propensity scores between the treatment and pseudo control groups.

**Figure S.1** Distribution of propensity scores in treatment and pseudo control groups before and after matching

*Notes:* Data are from the Socio-Economic Panel Survey (v36; unweighted; multiply imputed).

**Figure S.2** Distribution of propensity scores in treatment and pseudo control groups before and after matching

*Notes:* Data are from the Socio-Economic Panel Survey (v36; unweighted; multiply imputed).

**Table S.2** Means and standard deviations of covariates measured in the matching year of the divorce sample and the control sample. Before and after matching

| Covariates | Divorce sample  (n = 1,127) | |  | Unmatched control sample  (n = 19,604) | | Standardized difference in means (Cohen’s *d*) | Pseudo control group  (n = 3,883) | | Standardized difference in means  (Cohen’s *d*) |
| --- | --- | --- | --- | --- | --- | --- | --- | --- | --- |
|  | *M* | *SD* |  | *M* | *SD* |  | *M* | *SD* |  |
| **Basic demographics** |  |  |  |  |  |  |  |  |  |
| Female | 0.56 | 0.50 |  | 0.52 | 0.50 | -0.09 | 0.57 | 0.49 | 0.01 |
| Age | 31.59 | 7.30 |  | 38.57 | 9.47 | 0.74 | 31.39 | 7.14 | -0.03 |
| Cohort | 2.44 | 0.93 |  | 2.51 | 1.14 | 0.05 | 2.48 | 0.89 | 0.04 |
| Migration background | 0.16 | 0.36 |  | 0.27 | 0.44 | 0.26 | 0.17 | 0.38 | 0.04 |
| **Family of origin** |  |  |  |  |  |  |  |  |  |
| Number of siblings | 1.91 | 1.89 |  | 1.91 | 1.85 | 0.00 | 1.94 | 1.78 | 0.02 |
| Parents’ highest level of education | 0.94 | 0.55 |  | 0.96 | 0.58 | 0.04 | 0.93 | 0.55 | -0.01 |
| **Living arrangements** |  |  |  |  |  |  |  |  |  |
| Number of household members aged 0 to 17 years^*^ | 1.11 | 1.06 |  | 1.25 | 1.15 | 0.13 | 1.12 | 1.06 | 0.01 |
| Number of household members aged 18 years and over^*^ | 2.11 | 0.41 |  | 2.23 | 0.55 | 0.22 | 2.12 | 0.42 | 0.01 |
| Currently living in Eastern German federal state^*^ | 0.23 | 0.42 |  | 0.20 | 0.40 | -0.09 | 0.23 | 0.42 | -0.01 |
| **Health status** |  |  |  |  |  |  |  |  |  |
| Household member needs care/assistance^*^ | 0.01 | 0.10 |  | 0.02 | 0.15 | 0.08 | 0.01 | 0.11 | 0.03 |
| **Human capital & financial situation** |  |  |  |  |  |  |  |  |  |
| Educational level | 1.08 | 0.59 |  | 1.20 | 0.63 | 0.19 | 1.11 | 0.59 | 0.06 |
| Personal earnings (log) | 8.22 | 3.77 |  | 8.21 | 3.96 | 0.00 | 8.12 | 3.85 | -0.03 |
| Equalized household post-government income (log) | 9.88 | 0.48 |  | 9.99 | 0.52 | 0.23 | 9.90 | 0.44 | 0.06 |
| Employment status | 0.74 | 0.91 |  | 0.74 | 0.89 | -0.01 | 0.76 | 0.91 | 0.02 |
| Number of years in full-time work | 7.84 | 6.79 |  | 11.99 | 9.71 | 0.43 | 7.62 | 6.54 | -0.03 |
| Satisfaction with household income | 5.98 | 2.52 |  | 6.76 | 2.26 | 0.34 | 6.20 | 2.31 | 0.09 |
| Worries about own financial situation | 0.96 | 0.69 |  | 1.10 | 0.70 | 0.20 | 0.99 | 0.69 | 0.05 |
| **Wealth** |  |  |  |  |  |  |  |  |  |
| Homeownership^*^ | 0.26 | 0.44 |  | 0.47 | 0.50 | 0.42 | 0.30 | 0.46 | 0.07 |
| Savings account ownership^*^ | 0.74 | 0.44 |  | 0.73 | 0.45 | -0.03 | 0.76 | 0.43 | 0.05 |
| Ownership of business assets^*^ | 0.05 | 0.22 |  | 0.07 | 0.25 | 0.07 | 0.05 | 0.22 | 0.00 |
| Holding building loan^*^ | 0.52 | 0.50 |  | 0.53 | 0.50 | 0.01 | 0.54 | 0.50 | 0.03 |
| Life insurance^*^ | 0.65 | 0.48 |  | 0.64 | 0.48 | -0.01 | 0.66 | 0.47 | 0.03 |
| Ownership of shares^*^ | 0.16 | 0.37 |  | 0.18 | 0.38 | 0.05 | 0.18 | 0.39 | 0.06 |
| Capital gains^*^ | 1.00 | 0.84 |  | 1.21 | 0.95 | 0.23 | 1.07 | 0.87 | 0.08 |
| **Partner-level characteristics** |  |  |  |  |  |  |  |  |  |
| Partner’s age | 32.19 | 7.77 |  | 39.13 | 10.02 | 0.70 | 32.25 | 7.69 | 0.01 |
| Partner’s migration background | 0.17 | 0.37 |  | 0.27 | 0.44 | 0.22 | 0.18 | 0.39 | 0.04 |
| Partner’s number of siblings | 1.63 | 1.84 |  | 1.89 | 1.84 | 0.14 | 1.73 | 1.57 | 0.06 |
| Partner’s parents’ highest level of education | 0.93 | 0.55 |  | 0.96 | 0.57 | 0.06 | 0.92 | 0.55 | -0.02 |
| Partner’s educational level | 1.06 | 0.58 |  | 1.20 | 0.63 | 0.22 | 1.10 | 0.59 | 0.07 |
| Partner’s employment status | 0.63 | 0.89 |  | 0.73 | 0.89 | 0.12 | 0.58 | 0.87 | -0.06 |
| Partner’s number of years in full-time work | 9.00 | 7.62 |  | 12.87 | 10.49 | 0.37 | 9.10 | 7.47 | 0.01 |
| Partner’s satisfaction with household income | 5.93 | 2.47 |  | 6.73 | 2.26 | 0.35 | 6.17 | 2.31 | 0.10 |
| Partner’s worries about own financial situation | 0.97 | 0.68 |  | 1.10 | 0.70 | 0.19 | 1.00 | 0.68 | 0.05 |

*Notes:* Data are from the Socio-Economic Panel Survey (v36; imputed and unweighted). ^*^Variables measured at the household level. See Table S.1 for information on the variable type and the categories of discrete variables.

# Overview of SOEP Wealth Data

Since 2002, the SOEP survey team has been collecting comprehensive wealth data on a quadrennial basis (2002, 2007, 2012, and 2017). The wealth module covers a total of nine asset and liability components. Information on asset components is collected for financial assets, business assets, tangible assets, owner-occupied housing, other property, and private pensions and life insurances. The debt components include mortgage debts for owner-occupied property, mortgage on other property, and consumer credits.

Whereas other panel studies commonly measure the majority of wealth components at the household-level, with one household member providing information on the financial standing of the entire household, all wealth information is collected at the individual-level within the SOEP. This means that each household member 17 years of age or older is surveyed about their personal and potentially shared assets and liabilities.

Wealth data collection follows several steps: (1) a filter question (yes/no) is asked to assess whether the respondent personally holds a certain type of asset or liability. (2) if respondents answer in the affirmative, they are asked to provide the total value of the asset or liability. (3) a second filter question (yes/no) is asked to assess whether the asset or liability is held jointly. This is only done for wealth components that can theoretically be owned jointly (e.g., housing equity). (4) if respondents affirm joint ownership, they are asked to provide their personal share in percentage points. Using the total metric value of wealth components and their personal shares, the SOEP team calculates the value of personally owned assets and liabilities. Based on all household members’ personal wealth, the SOEP team further aggregates personal-level wealth to the household-level.

# Supplementary Table

**Table S.3** Descriptive statistics for imputed data.

|  | Divorced | | | Married | | |
| --- | --- | --- | --- | --- | --- | --- |
|  | Overall | Men | Women | Overall | Men | Women |
|  | mean/ (SE) | mean/ (SE) | mean/ (SE) | mean/ (SE) | mean/ (SE) | mean/ (SE) |
| **Basic demographics** |  |  |  |  |  |  |
| Age | 45.66 | 47.06 | 44.50 | 44.96 | 45.88 | 44.23 |
|  | 7.77 | 7.21 | 8.02 | 7.73 | 7.40 | 7.90 |
| Migration background | (0.13) | (0.11) | (0.14) | (0.13) | (0.12) | (0.13) |
| **Marital status** |  |  |  |  |  |  |
| Age at first marriage | 25.37 | 26.94 | 24.07 | 24.70 | 25.86 | 23.78 |
|  | (5.11) | (4.95) | (4.87) | (4.45) | (4.32) | (4.33) |
| Age at divorce | 38.53 | 39.81 | 37.48 | 37.42 | 38.14 | 36.85 |
|  | (7.37) | (6.92) | (7.57) | (7.07) | (6.95) | (7.12) |
| Ever remarried after divorce | 0.37 | 0.41 | 0.34 | 0.00 | 0.00 | 0.00 |
| **Family of origin** |  |  |  |  |  |  |
| Number of siblings | 1.92 | 2.05 | 1.81 | 2.00 | 2.05 | 1.95 |
|  | (1.82) | (1.93) | (1.73) | (1.74) | (1.83) | (1.66) |
| Parents' educational level (based on ISCED97) |  |  |  |  |  |  |
| low | 0.18 | 0.17 | 0.18 | 0.18 | 0.18 | 0.17 |
| intermediate | 0.71 | 0.71 | 0.71 | 0.71 | 0.70 | 0.71 |
| high | 0.12 | 0.12 | 0.12 | 0.11 | 0.11 | 0.12 |
| **Living arrangements** |  |  |  |  |  |  |
| Number of household members aged 0-17 | 0.69 | 0.41 | 0.92 | 1.08 | 1.09 | 1.08 |
|  | (0.96) | (0.74) | (1.06) | (1.08) | (1.08) | (1.09) |
| Number of household members age >17 | 1.73 | 1.67 | 1.78 | 2.46 | 2.48 | 2.44 |
|  | (0.75) | (0.71) | (0.78) | (0.69) | (0.7(0) | (0.68) |
| Currently in East Germany | 0.23 | 0.23 | 0.24 | 0.26 | 0.25 | 0.26 |
| **Health status** |  |  |  |  |  |  |
| Household member requires help/care | 0.02 | 0.01 | 0.03 | 0.02 | 0.02 | 0.02 |
| **Human capital & financial situation** |  |  |  |  |  |  |
| Educational level (based on ISCED97) |  |  |  |  |  |  |
| low | 0.12 | 0.10 | 0.13 | 0.10 | 0.07 | 0.11 |
| intermediate | 0.61 | 0.60 | 0.62 | 0.60 | 0.59 | 0.62 |
| high | 0.28 | 0.30 | 0.26 | 0.30 | 0.34 | 0.27 |
| Individual Earnings (LOG) | 8.65 | 9.36 | 8.07 | 8.62 | 9.89 | 7.61 |
|  | (3.63) | (3.21) | (3.86) | (3.52) | (2.51) | (3.87) |
| Annual Equiv. HH Income (LOG) | 9.94 | 10.03 | 9.86 | 10.07 | 10.07 | 10.07 |
|  | (0.55) | (0.56) | (0.52) | (0.43) | (0.42) | (0.43) |
| Employment status |  |  |  |  |  |  |
| full-time | 0.60 | 0.81 | 0.42 | 0.53 | 0.89 | 0.25 |
| part-time | 0.17 | 0.03 | 0.29 | 0.23 | 0.02 | 0.39 |
| other | 0.23 | 0.16 | 0.28 | 0.24 | 0.10 | 0.36 |
| Full-time work experience (in years) | 16.65 | 22.77 | 11.58 | 15.40 | 22.46 | 9.77 |
|  | (10.37) | (8.44) | (8.99) | (10.49) | (8.34) | (8.43) |
| Satisfaction with household income | 5.78 | 5.91 | 5.67 | 6.49 | 6.35 | 6.61 |
|  | (2.44) | (2.37) | (2.50) | (2.07) | (2.09) | (2.06) |
| Financial concerns | 0.93 | 0.99 | 0.88 | 1.04 | 1.05 | 1.03 |
|  | (0.70) | (0.70) | (0.69) | (0.66) | (0.66) | (0.65) |
| **Wealth** |  |  |  |  |  |  |
| Personal wealth (1'000 EUR) | 68.80 | 84.43 | 55.87 | 107.91 | 124.48 | 94.70 |
|  | (175.48) | (208.53) | (141.25) | (215.32) | (267.28) | (161.21) |
| Homeowner | 0.36 | 0.37 | 0.34 | 0.66 | 0.67 | 0.65 |
| Owned savings account in last year | 0.59 | 0.58 | 0.60 | 0.74 | 0.73 | 0.75 |
| Held business assets in last year | 0.05 | 0.07 | 0.04 | 0.07 | 0.07 | 0.08 |
| Held building loan contract in last year | 0.36 | 0.39 | 0.34 | 0.56 | 0.56 | 0.57 |
| Owned life insurance in last year | 0.50 | 0.54 | 0.46 | 0.69 | 0.69 | 0.69 |
| Held shares in last year | 0.07 | 0.07 | 0.06 | 0.14 | 0.14 | 0.14 |
| Capital gains |  |  |  |  |  |  |
| none | 0.31 | 0.28 | 0.34 | 0.16 | 0.17 | 0.16 |
| under 250 EUR | 0.47 | 0.46 | 0.48 | 0.48 | 0.48 | 0.48 |
| 250 EUR to <1000 EUR | 0.15 | 0.16 | 0.13 | 0.23 | 0.23 | 0.23 |
| 1000 EUR and more | 0.07 | 0.10 | 0.06 | 0.13 | 0.12 | 0.13 |
| Observations | 2067 | 936 | 1131 | 7734 | 3431 | 4303 |
| Individuals | 1127 | 492 | 635 | 3892 | 1689 | 2203 |

*Notes:* Data are from the Socio-Economic Panel Survey (v36).

**Table S.4** Cell sizes across years since divorce with 0 being the year of divorce

| Years since divorce | Treatment group | | Pseudo control group | |
| --- | --- | --- | --- | --- |
|  | Women | Men | Women | Men |
| 0 | 125 | 92 | 360 | 219 |
| 1 | 125 | 94 | 353 | 307 |
| 2 | 99 | 76 | 383 | 267 |
| 3 | 91 | 79 | 283 | 200 |
| 4 | 101 | 66 | 277 | 221 |
| 5 | 69 | 74 | 318 | 222 |
| 6 | 68 | 60 | 292 | 270 |
| 7 | 60 | 47 | 257 | 216 |
| 8 | 53 | 44 | 201 | 159 |
| 9 | 45 | 37 | 196 | 168 |
| 10 | 38 | 39 | 185 | 137 |
| 11 | 40 | 42 | 205 | 177 |
| 12 | 29 | 37 | 154 | 141 |
| 13 | 42 | 25 | 134 | 133 |
| 14 | 26 | 22 | 113 | 104 |
| 15 | 22 | 24 | 117 | 76 |
| 16 | 14 | 21 | 116 | 87 |
| 17 | 8 | 15 | 77 | 67 |
| 18 | 20 | 5 | 57 | 69 |
| 19 | 14 | 10 | 32 | 48 |
| 20 | 14 | 9 | 48 | 35 |
| 21 | 5 | 7 | 41 | 34 |
| 22 | 3 | 2 | 27 | 18 |
| 23 | 8 | 2 | 25 | 19 |
| 24 | 6 | 4 | 11 | 14 |
| 25 | 3 | 0 | 11 | 7 |
| 26 | 1 | 2 | 13 | 8 |
| 27 | 0 | 0 | 6 | 4 |
| 28 | 0 | 0 | 7 | 3 |
| 29 | 1 | 1 | 0 | 1 |
| 30 | 1 | 0 | 1 | 0 |

*Notes:* Data are from the Socio-Economic Panel Survey (v36)

**Table S.5** Linear random-effects growth curve models of personal net wealth

|  | Overall | Remarriage interaction | Gender interaction |
| --- | --- | --- | --- |
|  | B/(SE) | B/(SE) | B/(SE) |
| Divorce duration (in years) | 3225.25*** | 3232.01*** | 3841.81*** |
|  | (510.89) | (511.85) | (700.18) |
| Divorced (Ref: continuously married) | -34660.81*** | -33403.48*** | -32945.91** |
|  | (7335.93) | (8426.27) | (11028.56) |
| Divorced X divorce duration | -737.14 | -1076.90 | -1701.05 |
|  | (1023.91) | (1243.12) | (1542.03) |
| Remarried (Ref: unmarried) |  | -3687.76 |  |
|  |  | (15111.20) |  |
| Remarried X divorce duration |  | 766.14 |  |
|  |  | (1961.08) |  |
| Female (Ref: male) |  |  | -21808.98** |
|  |  |  | (7305.71) |
| Female X divorce duration |  |  | -1369.30 |
|  |  |  | (877.28) |
| Female X divorced |  |  | -3816.60 |
|  |  |  | (14568.14) |
| Female X divorced X divorce duration |  |  | 1788.55 |
|  |  |  | (2087.25) |
| First wealth observation | -11670.24** | -11602.77** | -12387.69** |
|  | (3855.20) | (3894.90) | (3879.49) |
| Imp. wealth flag | 14506.41** | 14517.05** | 14719.35** |
|  | (4565.48) | (4552.39) | (4570.39) |
| 2012/2017 (Ref: 2002/2012) | -4928.89 | -4975.96 | -3943.70 |
|  | (4068.10) | (4051.36) | (4057.55) |
| Currently living in Eastern Germany | -33739.04*** | -33706.09*** | -33641.44*** |
|  | (6304.15) | (6304.29) | (6288.19) |
| Intercept | 94921.94*** | 94868.79*** | 107743.69*** |
|  | (4862.31) | (4879.51) | (6653.92) |
| *Variance components:* |  |  |  |
| Slope | 11419.83 | 11417.03 | 11392.23 |
|  | (609.72) | (608.98) | (613.81) |
| Intercept | 145333.40 | 145331.27 | 144863.22 |
|  | (7972.17) | (7974.51) | (8010.87) |
| Covariance | 0.08 | 0.08 | 0.08 |
|  | (0.07) | (0.07) | (0.07) |
| Residuals | 110274.88 | 110272.05 | 110317.21 |
|  | (6305.01) | (6307.78) | (6297.08) |
| N Observations | 9801 | 9801 | 9801 |
| N Individuals | 5019 | 5019 | 5019 |

*Notes:* Data are from the Socio-Economic Panel Survey v36 (2002, 2007, 2012, 2017), imputed and unweighted. * p<.05, ** p<.01, *** p<.001

# Supplementary Figures

**Figure S.3** Random-effects growth model: Predicted personal Housing wealth trajectories for divorcees and continuously married overall and including an interaction for remarriage

*Note:* Whiskers indicate 95% confidence intervals. Data are from the Socio-Economic Panel Survey v36 (2002, 2007, 2012, 2017; unweighted; multiply imputed).

**Figure S.4** Random-effects growth model: Predicted personal Housing wealth trajectories for divorcees and continuously married overall and including an interaction for remarriage

*Note:* Whiskers indicate 95% confidence intervals. Data are from the Socio-Economic Panel Survey v36 (2002, 2007, 2012, 2017; unweighted; multiply imputed).

**Figure S.5** Random-effects growth model: Predicted personal financial wealth trajectories for divorcees and continuously married overall and including an interaction for remarriage

*Note:* Whiskers indicate 95% confidence intervals. Data are from the Socio-Economic Panel Survey v36 (2002, 2007, 2012, 2017; unweighted; multiply imputed).

**Figure S.6** Random-effects growth model: Predicted personal financial wealth trajectories for divorcees and continuously married overall and including an interaction for remarriage

*Note:* Whiskers indicate 95% confidence intervals. Data are from the Socio-Economic Panel Survey v36 (2002, 2007, 2012, 2017; unweighted; multiply imputed).

**Figure S.7** Random-effects growth model: Predicted personal wealth trajectories for divorcees and continuously married overall and including an interaction for remarriage up to 30, 20 and 15 years after the year of divorce

*Note:* Whiskers indicate 95% confidence intervals. Data are from the Socio-Economic Panel Survey v34 (2002, 2007, 2012, 2017; unweighted; multiply imputed).

**Figure S.8** Random-effects growth model: Predicted personal wealth trajectories for divorcees and continuously married men and women up to 30, 20 and 15 years after the year of divorce

*Note:* Whiskers indicate 95% confidence intervals. Data are from the Socio-Economic Panel Survey v36 (2002, 2007, 2012, 2017; unweighted; multiply imputed).
